# Crystal structure of 4-((1*E*)-1-{(2*Z*)-2-[4-(4-bromo­phen­yl)-3-phenyl-2,3-di­hydro-1,3-thia­zol-2-yl­idene]hydrazin-1-yl­idene}eth­yl)phenol hemihydrate

**DOI:** 10.1107/S1600536814019473

**Published:** 2014-09-24

**Authors:** Joel T. Mague, Mehmet Akkurt, Shaaban K. Mohamed, Alaa A. Hassan, Mustafa R. Albayati

**Affiliations:** a Department of Chemistry, Tulane University, New Orleans, LA 70118, USA; b Department of Physics, Faculty of Sciences, Erciyes University, 38039 Kayseri, Turkey; c Chemistry and Environmental Division, Manchester Metropolitan University, Manchester M1 5GD, England; d Chemistry Department, Faculty of Science, Minia University, 61519 El-Minia, Egypt; e Chemistry Department, Faculty of Science, Mini University, 61519 El-Minia, Egypt; f Kirkuk University, College of Science, Department of Chemistry, Kirkuk, Iraq

**Keywords:** crystal structure, phenol, C—H⋯S inter­actions, thia­zole scaffold compounds, medicinal applications

## Abstract

In the title compound, C_23_H_18_BrN_3_OS·0.5H_2_O, the bromo­phenyl, phenyl and phenol rings make dihedral angles of 46.5 (1), 66.78 (8) and 15.4 (2)°, respectively, with the mean squares plane of the thia­zol­idene ring. In the crystal, the lattice water mol­ecule is hydrogen bonded to the phenol group and makes a weaker O—H⋯N connection to an inversion-related mol­ecule, forming a ring while weak pairwise C—H⋯S inter­actions involving inversion-related mol­ecules form a second ring. Both these motifs result in the formation of two-dimensional networks lying parallel to (10-1).

## Related literature   

For the wide spectrum of medicinal applications of thia­zole scaffold compounds, see: Pattan *et al.* (2009 ▶); Sharma *et al.* (2009 ▶); Argyropoulou *et al.* (2009 ▶); Trautman & Longe (1948 ▶); Surray (1949 ▶); Bhattacharya *et al.* (2005 ▶); Alemagna *et al.* (1968 ▶); Spector *et al.* (1998 ▶); Karade *et al.* (2008 ▶). For a related structure, see: Akkurt *et al.* (2014 ▶).
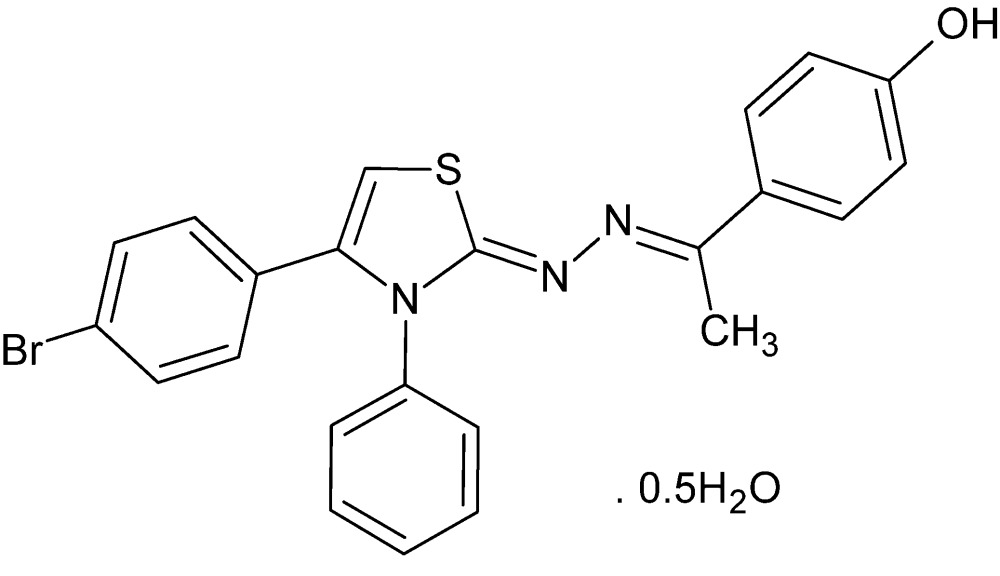


## Experimental   

### Crystal data  

C_23_H_18_BrN_3_OS·0.5H_2_O
*M* *_r_* = 473.38
Triclinic, 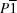

*a* = 8.485 (2) Å
*b* = 10.336 (2) Å
*c* = 12.057 (3) Å
α = 80.515 (3)°
β = 88.008 (3)°
γ = 86.249 (4)°
*V* = 1040.3 (4) Å^3^
*Z* = 2
Mo *K*α radiation
μ = 2.10 mm^−1^
*T* = 150 K
0.27 × 0.23 × 0.07 mm

### Data collection  

Bruker SMART APEX CCD diffractometer
Absorption correction: multi-scan (*SADABS*; Bruker, 2013 ▶) *T*_min_ = 0.51, *T*_max_ = 0.86
18957 measured reflections
5211 independent reflections
3834 reflections with *I* > 2σ(*I*)
*R*_int_ = 0.049

### Refinement  

*R*[*F* ^2^ > 2σ(*F* ^2^)] = 0.046
*wR*(*F* ^2^) = 0.126
*S* = 1.02
5211 reflections
273 parameters
H-atom parameters constrained
Δρ_max_ = 0.85 e Å^−3^
Δρ_min_ = −0.32 e Å^−3^

### 

Data collection: *APEX2* (Bruker, 2013 ▶); cell refinement: *SAINT* (Bruker, 2013 ▶); data reduction: *SAINT*; program(s) used to solve structure: *SHELXT* (Sheldrick, 2008 ▶); program(s) used to refine structure: *SHELXL2014* (Sheldrick, 2008 ▶); molecular graphics: *DIAMOND* (Brandenburg & Putz, 2012 ▶); software used to prepare material for publication: *SHELXTL* (Sheldrick, 2008 ▶).

## Supplementary Material

Crystal structure: contains datablock(s) global, I. DOI: 10.1107/S1600536814019473/qm2108sup1.cif

Structure factors: contains datablock(s) I. DOI: 10.1107/S1600536814019473/qm2108Isup2.hkl

Supporting information file. DOI: 10.1107/S1600536814019473/qm2108Isup3.cml

**Fig. 1.**
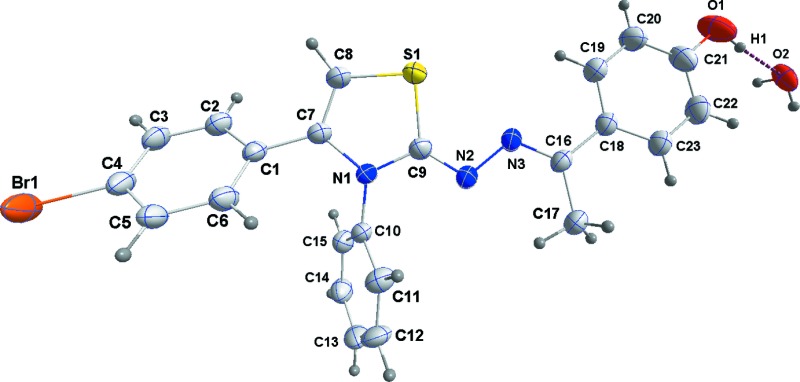
The title compound with numbering scheme and 50% probability displacement ellipsoids.

**Fig. 2.**
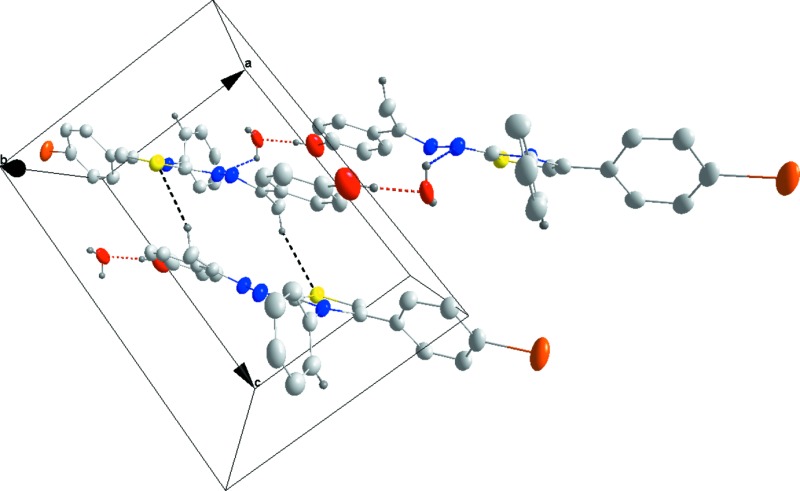
Packing viewed down the b axis showing the rings formed by the O—H···O (red), O—H···N (blue) and C—H···S (black) interactions.

. DOI: 10.1107/S1600536814019473/qm2108fig1.tif
The title compound with numbering scheme and 50% probability displacement ellipsoids.

b . DOI: 10.1107/S1600536814019473/qm2108fig2.tif
Packing viewed down the *b* axis showing the rings formed by the O—H⋯O (red), O—H⋯N (blue) and C—H⋯S (black) inter­actions.

CCDC reference: 1021529

Additional supporting information:  crystallographic information; 3D view; checkCIF report

## Figures and Tables

**Table 1:** Hydrogen-bond geometry (Å, °)

*D*—H⋯*A*	*D*—H	H⋯*A*	*D*⋯*A*	*D*—H⋯*A*
O1—H1⋯O2	0.84	1.67	2.511 (5)	173
O2—H2*A*⋯N2^i^	0.84	2.45	2.898 (5)	114
C17—H17*B*⋯S1^ii^	0.98	3.02	3.925 (3)	154

Symmetry codes: (i) 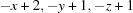; (ii) 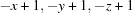.

## supplementary crystallographic information

### S1. Comment

Thiazole scaffold compounds are of considerable interest from therapeutic point of view due to their utility as antibacterial and antifungal (Pattan *et al*., 2009), anti-inflammatory (Sharma *et al.*, 2009), analgesic (Argyropoulou *et al.*, 2009), antitubercular (Trautman & Longe, 1948), central nervous system (*CNS*) stimulate (Surray, 1949), anti-HIV (Bhattacharya *et al.*, 2005), and algicidol (Alemagna *et al.*, 1968) activities. Some of thiazole derivatives have shown inhibition towards herpes simplex virus (Spector *et al.*, 1998). In addition, thiazole containing N=C=S moiety have been used as antiphychotics (Pattan *et al.*, 2009) and antimalarial (Karade *et al.*, 2008) agents. In this context and as part of our on-going study of bio-active heterocyclic molecules we herein report the synthesis and crystal structure of the title compound. In the title compound, the phenyl rings C1–C6, C10–C15 and C18–C23 make dihedral angles, respectively, of 46.5 (1), 66.78 (8) and 15.4 (2)° with the least squares plane of the thiazolidene ring. The lattice water is hydrogen bonded to the phenol group and makes a weaker connection to N2^i^ (i: 2-*x*, 1-*y*, 1-*z*) to form a ring while weak, pairwise C17—H17B···S1 interactions with the molecule at 1-*x*, 1-*y*, 1-*z* form a second ring (Fig. 2 and Table 1). Both these motifs extend along (101).

### S2. Experimental

The title compound has been prepared according to our reported method (Akkurt *et al.*, 2014). Mono-crystals suitable for X-ray diffraction have been obtained by crystallization of the crude product (I) from ethanol.

### S3. Refinement

H-atoms attached to carbon were placed in calculated positions (C—H = 0.95 - 0.98 Å) while those attached to oxygen were placed in locations derived from a difference map and their coordinates adjusted to give O—H = 0.84 Å. All were included as riding contributions with isotropic displacement parameters 1.2 - 1.5 times those of the attached atoms. The crystals of the title compound were quite small and very weakly diffracting.

### Crystal data

**Table:**

C_23_H_18_BrN_3_OS·0.5H_2_O	*Z* = 2
*M**_r_* = 473.38	*F*(000) = 482
Triclinic, *P*1	*D*_x_ = 1.511 Mg m^−^^3^
*a* = 8.485 (2) Å	Mo *K*α radiation, λ = 0.71073 Å
*b* = 10.336 (2) Å	Cell parameters from 7895 reflections
*c* = 12.057 (3) Å	θ = 2.4–28.4°
α = 80.515 (3)°	µ = 2.10 mm^−^^1^
β = 88.008 (3)°	*T* = 150 K
γ = 86.249 (4)°	Plate, orange
*V* = 1040.3 (4) Å^3^	0.27 × 0.23 × 0.07 mm

### Data collection

**Table:**

Bruker SMART APEX CCD diffractometer	5211 independent reflections
Radiation source: fine-focus sealed tube	3834 reflections with *I* > 2σ(*I*)
Graphite monochromator	*R*_int_ = 0.049
Detector resolution: 8.3660 pixels mm^-1^	θ_max_ = 28.5°, θ_min_ = 2.0°
φ and ω scans	*h* = −11→11
Absorption correction: multi-scan (*SADABS*; Bruker, 2013)	*k* = −13→13
*T*_min_ = 0.51, *T*_max_ = 0.86	*l* = −16→16
18957 measured reflections	

### Refinement

**Table:**

Refinement on *F*^2^	Primary atom site location: structure-invariant direct methods
Least-squares matrix: full	Secondary atom site location: difference Fourier map
*R*[*F*^2^ > 2σ(*F*^2^)] = 0.046	Hydrogen site location: mixed
*wR*(*F*^2^) = 0.126	H-atom parameters constrained
*S* = 1.02	*w* = 1/[σ^2^(*F*_o_^2^) + (0.062*P*)^2^ + 0.5209*P*] where *P* = (*F*_o_^2^ + 2*F*_c_^2^)/3
5211 reflections	(Δ/σ)_max_ < 0.001
273 parameters	Δρ_max_ = 0.85 e Å^−^^3^
0 restraints	Δρ_min_ = −0.32 e Å^−^^3^

### Special details

**Table:**

Experimental. The diffraction data were obtained from 3 sets of 400 frames, each of width 0.5° in ω, collected at φ = 0.00, 90.00 and 180.00° and 2 sets of 800 frames, each of width 0.45° in φ, collected at ω = -30.00 and 210.00°. The scan time was 15 sec/frame.
Geometry. All e.s.d.'s (except the e.s.d. in the dihedral angle between two l.s. planes) are estimated using the full covariance matrix. The cell e.s.d.'s are taken into account individually in the estimation of e.s.d.'s in distances, angles and torsion angles; correlations between e.s.d.'s in cell parameters are only used when they are defined by crystal symmetry. An approximate (isotropic) treatment of cell e.s.d.'s is used for estimating e.s.d.'s involving l.s. planes.
Refinement. Refinement of *F*^2^ against ALL reflections. The weighted *R*-factor *wR* and goodness of fit *S* are based on *F*^2^, conventional *R*-factors *R* are based on *F*, with *F* set to zero for negative *F*^2^. The threshold expression of *F*^2^ > σ(*F*^2^) is used only for calculating *R*-factors(gt) *etc*. and is not relevant to the choice of reflections for refinement. *R*-factors based on *F*^2^ are statistically about twice as large as those based on *F*, and *R*- factors based on ALL data will be even larger. H-atoms attached to carbon were placed in calculated positions (C—H = 0.95 - 0.98 Å) while those attached to oxygen were placed in locations derived from a difference map and their coordinates adjusted to give O—H = 0.84 Å. All were included as riding contributions with isotropic displacement parameters 1.2 - 1.5 times those of the attached atoms.

### Fractional atomic coordinates and isotropic or equivalent isotropic displacement parameters (Å^2^)

**Table:**

	*x*	*y*	*z*	*U*_iso_*/*U*_eq_	Occ. (<1)
Br1	−0.12028 (5)	0.13286 (3)	−0.19996 (3)	0.05610 (15)	
S1	0.40464 (9)	0.58630 (7)	0.18323 (6)	0.03452 (17)	
O1	0.9721 (3)	0.8848 (3)	0.5879 (3)	0.0730 (8)	
H1	1.0394	0.8562	0.6368	0.110*	
N1	0.3970 (3)	0.3403 (2)	0.16873 (18)	0.0301 (5)	
N2	0.5690 (3)	0.3796 (2)	0.3070 (2)	0.0369 (5)	
N3	0.6250 (3)	0.4875 (2)	0.34845 (19)	0.0354 (5)	
C1	0.1997 (3)	0.3384 (3)	0.0175 (2)	0.0290 (5)	
C2	0.1835 (3)	0.3850 (3)	−0.0973 (2)	0.0347 (6)	
H2	0.2382	0.4593	−0.1313	0.042*	
C3	0.0891 (3)	0.3245 (3)	−0.1624 (2)	0.0370 (6)	
H3	0.0798	0.3562	−0.2406	0.044*	
C4	0.0090 (3)	0.2178 (3)	−0.1119 (2)	0.0374 (6)	
C5	0.0177 (3)	0.1714 (3)	0.0027 (2)	0.0357 (6)	
H5	−0.0417	0.0999	0.0366	0.043*	
C6	0.1144 (3)	0.2311 (3)	0.0667 (2)	0.0343 (6)	
H6	0.1231	0.1990	0.1449	0.041*	
C7	0.2959 (3)	0.4075 (3)	0.0851 (2)	0.0296 (5)	
C8	0.2881 (3)	0.5384 (3)	0.0827 (2)	0.0336 (6)	
H8	0.2256	0.5985	0.0314	0.040*	
C9	0.4677 (3)	0.4211 (3)	0.2289 (2)	0.0319 (6)	
C10	0.4530 (3)	0.2040 (3)	0.1771 (2)	0.0304 (5)	
C11	0.4261 (3)	0.1177 (3)	0.2750 (3)	0.0396 (7)	
H11	0.3673	0.1464	0.3359	0.048*	
C12	0.4863 (4)	−0.0122 (3)	0.2834 (3)	0.0457 (8)	
H12	0.4673	−0.0727	0.3501	0.055*	
C13	0.5730 (4)	−0.0532 (3)	0.1955 (3)	0.0460 (8)	
H13	0.6136	−0.1419	0.2018	0.055*	
C14	0.6010 (4)	0.0336 (3)	0.0988 (3)	0.0426 (7)	
H14	0.6619	0.0052	0.0388	0.051*	
C15	0.5403 (3)	0.1627 (3)	0.0889 (2)	0.0369 (6)	
H15	0.5586	0.2227	0.0217	0.044*	
C16	0.6881 (3)	0.4600 (3)	0.4462 (2)	0.0321 (6)	
C17	0.6960 (4)	0.3259 (3)	0.5167 (2)	0.0383 (6)	
H17A	0.6467	0.2643	0.4769	0.058*	
H17B	0.6399	0.3296	0.5886	0.058*	
H17C	0.8067	0.2963	0.5305	0.058*	
C18	0.7578 (3)	0.5707 (3)	0.4874 (2)	0.0332 (6)	
C19	0.7384 (4)	0.6989 (3)	0.4292 (3)	0.0410 (7)	
H19	0.6745	0.7152	0.3646	0.049*	
C20	0.8087 (4)	0.8027 (3)	0.4621 (3)	0.0483 (8)	
H20	0.7939	0.8886	0.4201	0.058*	
C21	0.9007 (4)	0.7810 (3)	0.5567 (3)	0.0483 (8)	
C22	0.9198 (4)	0.6557 (3)	0.6184 (3)	0.0455 (7)	
H22	0.9815	0.6406	0.6840	0.055*	
C23	0.8486 (3)	0.5521 (3)	0.5839 (2)	0.0379 (6)	
H23	0.8620	0.4666	0.6270	0.045*	
O2	1.1835 (4)	0.8167 (5)	0.7301 (4)	0.0451 (11)	0.5
H2A	1.2451	0.8144	0.6743	0.054*	0.5
H2B	1.1914	0.7404	0.7668	0.054*	0.5

### Atomic displacement parameters (Å^2^)

**Table:**

	*U*^11^	*U*^22^	*U*^33^	*U*^12^	*U*^13^	*U*^23^
Br1	0.0777 (3)	0.0379 (2)	0.0543 (2)	−0.00352 (16)	−0.03883 (18)	−0.00462 (14)
S1	0.0432 (4)	0.0256 (3)	0.0347 (4)	−0.0034 (3)	−0.0061 (3)	−0.0030 (3)
O1	0.0642 (17)	0.0498 (16)	0.114 (3)	−0.0017 (13)	−0.0168 (16)	−0.0361 (16)
N1	0.0385 (12)	0.0249 (11)	0.0263 (11)	−0.0011 (9)	−0.0074 (9)	−0.0010 (8)
N2	0.0451 (13)	0.0337 (13)	0.0319 (12)	−0.0062 (10)	−0.0093 (10)	−0.0017 (10)
N3	0.0430 (13)	0.0340 (13)	0.0296 (12)	−0.0060 (10)	−0.0082 (10)	−0.0032 (9)
C1	0.0294 (12)	0.0261 (13)	0.0301 (13)	0.0052 (10)	−0.0044 (10)	−0.0028 (10)
C2	0.0343 (14)	0.0335 (15)	0.0332 (14)	0.0016 (11)	−0.0020 (11)	0.0018 (11)
C3	0.0408 (15)	0.0381 (16)	0.0303 (14)	0.0058 (12)	−0.0102 (12)	−0.0014 (11)
C4	0.0424 (15)	0.0304 (15)	0.0404 (15)	0.0074 (12)	−0.0170 (12)	−0.0091 (12)
C5	0.0437 (15)	0.0242 (13)	0.0386 (15)	−0.0005 (11)	−0.0093 (12)	−0.0019 (11)
C6	0.0433 (15)	0.0288 (14)	0.0294 (13)	−0.0003 (11)	−0.0054 (11)	−0.0008 (10)
C7	0.0319 (13)	0.0263 (13)	0.0292 (13)	−0.0002 (10)	−0.0031 (10)	−0.0003 (10)
C8	0.0357 (14)	0.0272 (14)	0.0367 (15)	0.0005 (11)	−0.0076 (11)	−0.0007 (11)
C9	0.0386 (14)	0.0285 (13)	0.0278 (13)	−0.0039 (11)	−0.0016 (11)	−0.0012 (10)
C10	0.0319 (13)	0.0246 (13)	0.0338 (14)	−0.0020 (10)	−0.0096 (11)	0.0004 (10)
C11	0.0404 (15)	0.0365 (16)	0.0384 (16)	0.0003 (12)	0.0003 (13)	0.0029 (12)
C12	0.0494 (17)	0.0319 (16)	0.0494 (18)	0.0003 (13)	−0.0087 (14)	0.0127 (13)
C13	0.0480 (17)	0.0315 (16)	0.058 (2)	0.0078 (13)	−0.0207 (15)	−0.0061 (14)
C14	0.0451 (17)	0.0420 (17)	0.0427 (17)	0.0022 (13)	−0.0085 (13)	−0.0134 (13)
C15	0.0424 (15)	0.0361 (15)	0.0322 (14)	−0.0024 (12)	−0.0048 (12)	−0.0047 (11)
C16	0.0351 (13)	0.0347 (15)	0.0245 (13)	0.0028 (11)	−0.0026 (10)	−0.0005 (10)
C17	0.0488 (16)	0.0353 (15)	0.0297 (14)	0.0007 (13)	−0.0087 (12)	−0.0013 (11)
C18	0.0321 (13)	0.0376 (15)	0.0294 (13)	0.0011 (11)	−0.0031 (11)	−0.0047 (11)
C19	0.0455 (16)	0.0392 (17)	0.0382 (16)	0.0023 (13)	−0.0087 (13)	−0.0063 (13)
C20	0.0512 (18)	0.0372 (17)	0.057 (2)	0.0005 (14)	−0.0099 (15)	−0.0074 (14)
C21	0.0410 (16)	0.0482 (19)	0.061 (2)	0.0012 (14)	−0.0070 (15)	−0.0239 (16)
C22	0.0374 (15)	0.058 (2)	0.0430 (17)	0.0024 (14)	−0.0106 (13)	−0.0152 (15)
C23	0.0360 (14)	0.0442 (17)	0.0332 (15)	0.0004 (12)	−0.0038 (12)	−0.0057 (12)
O2	0.0275 (19)	0.060 (3)	0.057 (3)	0.0017 (18)	−0.0138 (18)	−0.035 (2)

### Geometric parameters (Å, º)

**Table:**

Br1—C4	1.902 (3)	C11—C12	1.394 (4)
S1—C8	1.743 (3)	C11—H11	0.9500
S1—C9	1.760 (3)	C12—C13	1.377 (5)
O1—C21	1.376 (4)	C12—H12	0.9500
O1—H1	0.8400	C13—C14	1.372 (5)
N1—C9	1.371 (3)	C13—H13	0.9500
N1—C7	1.409 (3)	C14—C15	1.387 (4)
N1—C10	1.446 (3)	C14—H14	0.9500
N2—C9	1.297 (4)	C15—H15	0.9500
N2—N3	1.411 (3)	C16—C18	1.484 (4)
N3—C16	1.293 (3)	C16—C17	1.500 (4)
C1—C2	1.397 (4)	C17—H17A	0.9800
C1—C6	1.401 (4)	C17—H17B	0.9800
C1—C7	1.467 (4)	C17—H17C	0.9800
C2—C3	1.385 (4)	C18—C19	1.396 (4)
C2—H2	0.9500	C18—C23	1.397 (4)
C3—C4	1.377 (4)	C19—C20	1.380 (5)
C3—H3	0.9500	C19—H19	0.9500
C4—C5	1.388 (4)	C20—C21	1.384 (5)
C5—C6	1.385 (4)	C20—H20	0.9500
C5—H5	0.9500	C21—C22	1.386 (5)
C6—H6	0.9500	C22—C23	1.391 (4)
C7—C8	1.346 (4)	C22—H22	0.9500
C8—H8	0.9500	C23—H23	0.9500
C10—C11	1.379 (4)	O2—H2A	0.8397
C10—C15	1.384 (4)	O2—H2B	0.8384
			
C8—S1—C9	90.06 (13)	C13—C12—C11	120.4 (3)
C21—O1—H1	109.5	C13—C12—H12	119.8
C9—N1—C7	114.0 (2)	C11—C12—H12	119.8
C9—N1—C10	119.9 (2)	C14—C13—C12	120.2 (3)
C7—N1—C10	124.7 (2)	C14—C13—H13	119.9
C9—N2—N3	109.8 (2)	C12—C13—H13	119.9
C16—N3—N2	115.3 (2)	C13—C14—C15	120.0 (3)
C2—C1—C6	118.6 (2)	C13—C14—H14	120.0
C2—C1—C7	119.8 (2)	C15—C14—H14	120.0
C6—C1—C7	121.6 (2)	C10—C15—C14	119.8 (3)
C3—C2—C1	121.1 (3)	C10—C15—H15	120.1
C3—C2—H2	119.5	C14—C15—H15	120.1
C1—C2—H2	119.5	N3—C16—C18	116.0 (2)
C4—C3—C2	118.9 (3)	N3—C16—C17	124.2 (3)
C4—C3—H3	120.6	C18—C16—C17	119.8 (2)
C2—C3—H3	120.6	C16—C17—H17A	109.5
C3—C4—C5	121.8 (3)	C16—C17—H17B	109.5
C3—C4—Br1	119.5 (2)	H17A—C17—H17B	109.5
C5—C4—Br1	118.7 (2)	C16—C17—H17C	109.5
C6—C5—C4	118.9 (3)	H17A—C17—H17C	109.5
C6—C5—H5	120.6	H17B—C17—H17C	109.5
C4—C5—H5	120.6	C19—C18—C23	116.8 (3)
C5—C6—C1	120.8 (3)	C19—C18—C16	121.0 (2)
C5—C6—H6	119.6	C23—C18—C16	122.2 (3)
C1—C6—H6	119.6	C20—C19—C18	122.3 (3)
C8—C7—N1	112.4 (2)	C20—C19—H19	118.9
C8—C7—C1	125.0 (2)	C18—C19—H19	118.9
N1—C7—C1	122.4 (2)	C19—C20—C21	119.7 (3)
C7—C8—S1	113.1 (2)	C19—C20—H20	120.2
C7—C8—H8	123.5	C21—C20—H20	120.2
S1—C8—H8	123.5	O1—C21—C20	119.2 (3)
N2—C9—N1	123.9 (2)	O1—C21—C22	121.0 (3)
N2—C9—S1	125.6 (2)	C20—C21—C22	119.8 (3)
N1—C9—S1	110.52 (19)	C21—C22—C23	119.9 (3)
C11—C10—C15	120.5 (3)	C21—C22—H22	120.1
C11—C10—N1	119.8 (2)	C23—C22—H22	120.1
C15—C10—N1	119.6 (2)	C22—C23—C18	121.5 (3)
C10—C11—C12	119.1 (3)	C22—C23—H23	119.2
C10—C11—H11	120.5	C18—C23—H23	119.2
C12—C11—H11	120.5	H2A—O2—H2B	104.8
			
C9—N2—N3—C16	160.3 (2)	C8—S1—C9—N1	−1.1 (2)
C6—C1—C2—C3	−1.9 (4)	C9—N1—C10—C11	70.7 (3)
C7—C1—C2—C3	−177.8 (2)	C7—N1—C10—C11	−123.7 (3)
C1—C2—C3—C4	0.8 (4)	C9—N1—C10—C15	−105.9 (3)
C2—C3—C4—C5	1.5 (4)	C7—N1—C10—C15	59.7 (4)
C2—C3—C4—Br1	−179.6 (2)	C15—C10—C11—C12	−0.8 (4)
C3—C4—C5—C6	−2.5 (4)	N1—C10—C11—C12	−177.5 (3)
Br1—C4—C5—C6	178.6 (2)	C10—C11—C12—C13	0.8 (5)
C4—C5—C6—C1	1.3 (4)	C11—C12—C13—C14	0.0 (5)
C2—C1—C6—C5	0.8 (4)	C12—C13—C14—C15	−0.7 (5)
C7—C1—C6—C5	176.6 (2)	C11—C10—C15—C14	0.1 (4)
C9—N1—C7—C8	−0.9 (3)	N1—C10—C15—C14	176.8 (3)
C10—N1—C7—C8	−167.2 (2)	C13—C14—C15—C10	0.7 (4)
C9—N1—C7—C1	−175.1 (2)	N2—N3—C16—C18	175.8 (2)
C10—N1—C7—C1	18.5 (4)	N2—N3—C16—C17	−3.3 (4)
C2—C1—C7—C8	46.9 (4)	N3—C16—C18—C19	7.3 (4)
C6—C1—C7—C8	−128.9 (3)	C17—C16—C18—C19	−173.5 (3)
C2—C1—C7—N1	−139.6 (3)	N3—C16—C18—C23	−171.0 (3)
C6—C1—C7—N1	44.6 (4)	C17—C16—C18—C23	8.1 (4)
N1—C7—C8—S1	0.0 (3)	C23—C18—C19—C20	1.9 (4)
C1—C7—C8—S1	174.1 (2)	C16—C18—C19—C20	−176.5 (3)
C9—S1—C8—C7	0.6 (2)	C18—C19—C20—C21	−0.7 (5)
N3—N2—C9—N1	177.1 (2)	C19—C20—C21—O1	179.1 (3)
N3—N2—C9—S1	−1.5 (3)	C19—C20—C21—C22	−0.9 (5)
C7—N1—C9—N2	−177.5 (3)	O1—C21—C22—C23	−178.9 (3)
C10—N1—C9—N2	−10.4 (4)	C20—C21—C22—C23	1.0 (5)
C7—N1—C9—S1	1.3 (3)	C21—C22—C23—C18	0.3 (5)
C10—N1—C9—S1	168.41 (19)	C19—C18—C23—C22	−1.7 (4)
C8—S1—C9—N2	177.7 (3)	C16—C18—C23—C22	176.7 (3)

### Hydrogen-bond geometry (Å, º)

**Table:**

*D*—H···*A*	*D*—H	H···*A*	*D*···*A*	*D*—H···*A*
O1—H1···O2	0.84	1.67	2.511 (5)	173
O2—H2*A*···N2^i^	0.84	2.45	2.898 (5)	114
C17—H17*B*···S1^ii^	0.98	3.02	3.925 (3)	154

Symmetry codes: (i) −*x*+2, −*y*+1, −*z*+1; (ii) −*x*+1, −*y*+1, −*z*+1.
