# Supplementary figures and images for: Crystal structure of 4-((1E)-1-{(2Z)-2-[4-(4-bromo­phen­yl)-3-phenyl-2,3-di­hydro-1,3-thia­zol-2-yl­idene]hydrazin-1-yl­idene}eth­yl)phenol hemihydrate
Source: Acta Crystallogr Sect E Struct Rep Online. 2014 Sep 24;70(Pt 10):o1124–5. doi: 10.1107/S1600536814019473 (PMC4257180; doi:10.1107/S1600536814019473)

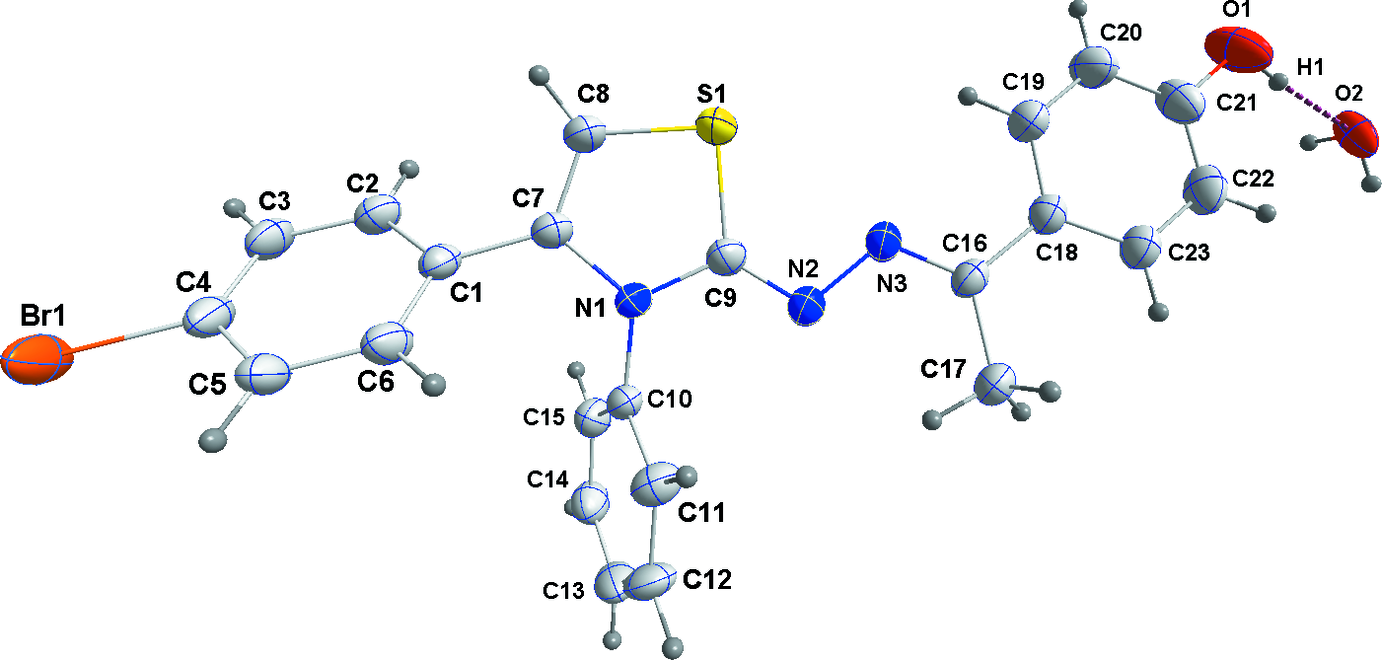

Supplement: Supplementary file 4 [file e-70-o1124-fig1.tif]

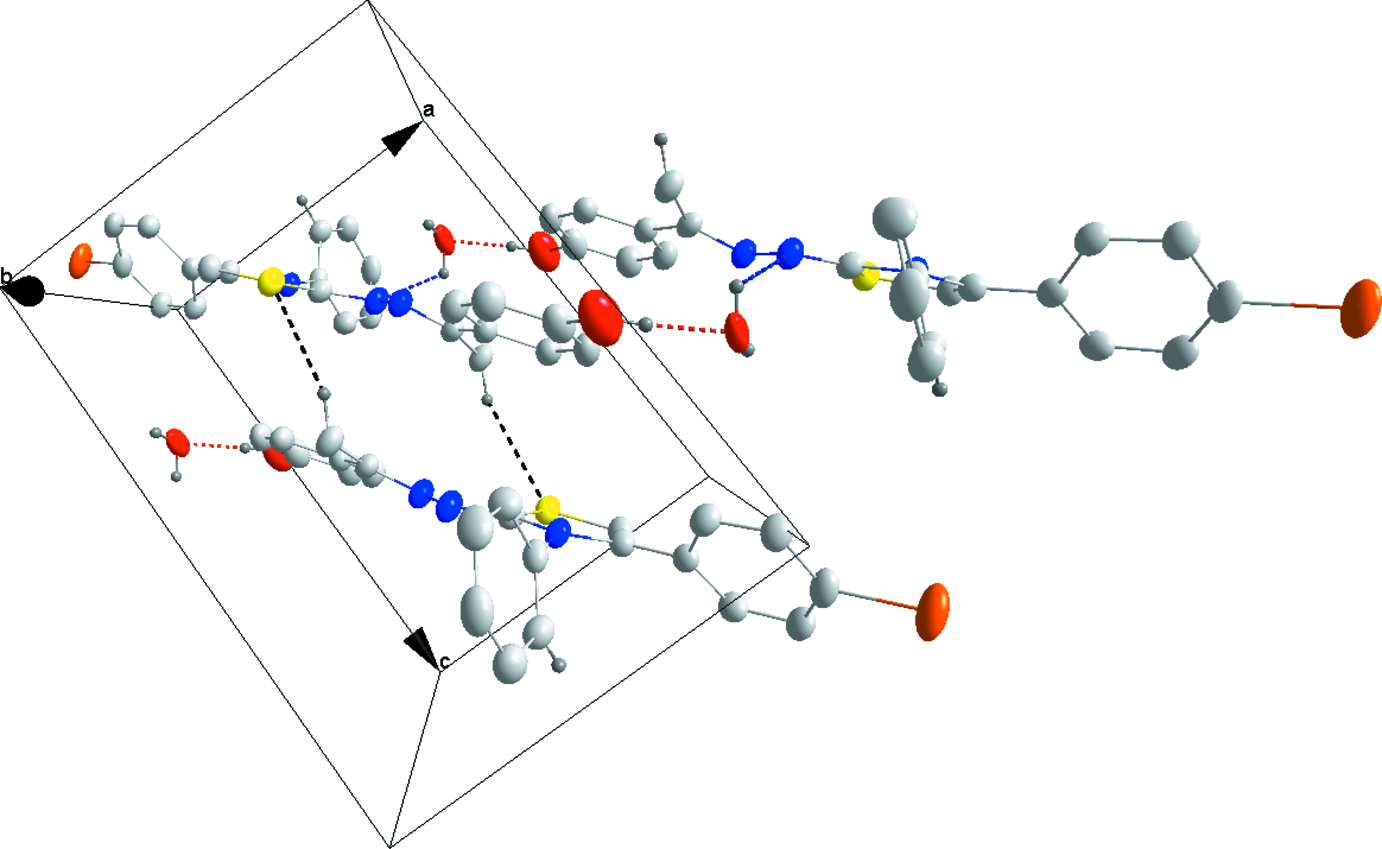

Supplement: Supplementary file 5 [file e-70-o1124-fig2.tif]
